# Comparison of Biogenic Amines in Chinese Commercial Soy Sauces

**DOI:** 10.3390/molecules24081522

**Published:** 2019-04-17

**Authors:** Jia Li, Linan Zhou, Wei Feng, Huan Cheng, Aliyu Idris Muhammad, Xingqian Ye, Zijian Zhi

**Affiliations:** 1 Life Science College, China Jiliang University, Hangzhou 310018 and China; hobo-scitech@139.com; 2 Zhejiang Wuweihe Food Co. Ltd., Hangzhou 313213, China; zhouln002@sina.com (L.Z.); fengwei-1982@163.com (W.F.); 3 School of Biosystems Engineering and Food Science, Zhejiang University, Hangzhou 310058, China; huancheng@zju.edu.cn (H.C.); aimuhammad.age@buk.edu.ng (A.I.M.); psu@zju.edu.cn (X.Y.); 4 Hobo Agricultural Sci-tech Co. Ltd., Hangzhou 310018, China

**Keywords:** biogenic amines, soy sauce, HPLC, comparison

## Abstract

Soy sauce contains a series of biogenic amines (BAs) which is a kind of bioactive organics relating to food quality and safety. High concentration of BAs may lead to remarkable physiological and toxicological influences on human bodies, including hypotension, dizziness, and headaches. Here, we systematically compared the levels of ten main BAs among 53 Chinese commercial soy sauces using an improved high-performance liquid chromatography (HPLC) method. The results showed that the brands and production regions were both important factors accounting for the BAs’ content. The contents of Cad, Spm, Try, Phe, His, and Tyr in dark soy sauces were higher than those in light soy sauces. His and Phe in dark soy sauces were 3.7 and 1.84 times higher than in light samples, respectively. Besides, it was surprising that the content of BAs in soy sauces hugely varied from place to place. This work comprehensively compared the content of BAs in soy sauces, showing the relation between soy sauce processes and BAs, offering abundant information for further research on BAs control.

## 1. Introduction

Soy sauce, a traditional fermented condiment, is generally prepared by months of enzymatic brewing of soy bean and wheat flour, which can be used to enhance the flavor and taste of foods [1]. Presently, it is popular all over the world, especially most Asian countries, mainly because of the increased consumption of oriental foods. However, due to the presence of microorganism and protein hydrolysis, microbial decarboxylation of amino acids or transamination of aldehydes would occur during the manufacturing process of soy sauce, resulting in the formation of undesirable compounds, such as biogenic amines (BAs) [2,3]. 

BAs are basic nitrogenous organic compounds with low molecular weights [4] that include tyramine (Tyr), 2-phenylethylamine (Phe), agmatine (Agm), histamine (His), serotonin (Ser), spermidine (Spd), putrescine (Put), cadaverine (Cad), tryptamine (Try) and spermine (Spm) [5]. It is significant to measure the contents of BAs in soy sauce since they are recognized as indicators of the quality and safety of products. The levels of certain amines in soy sauce can mirror the hygienic-sanitary conditions during the processing and quality of raw materials [3]. In addition, low levels of BAs play an essential role in humans and animals, including gastric acid secretion and body temperature regulation. The consumption of food containing high contents of BAs tends to exert the toxicological and organoleptic effects, such as His, Tyr, Try, and Phe. These effects are likely to cause some intoxication symptoms of hypotension, nausea, palpitations, rashes, dizziness, headaches, tachycardia, hypertension, cardiac, and emesis [4,6]. Furthermore, BAs are also considered as precursors of carcinogens, e.g., N-nitrosamines [7]. Therefore, the BA concentration in food should be reasonably controlled.

At present, there are few biogenic amines limits on food, and this is mainly due to the fact that the toxicological threshold varies because of individual differences and different populations. According to their sensitivity, the toxic dose may be from several mg/kg to several hundred mg/kg [8]. Thus, some countries, such as the USA, Sweden, Austria and the Netherlands, have established regulations and legal requirements for the maximum limits of biogenic amines (mainly His) in various foods. The US Food and Drug Administration (FDA) regulated the maximum histamine level in fishery products at 50 mg/kg [9] whereas some countries in Europe recommended establishing limits for histamine in wine [Germany (2 mg/L), Belgium (5–6 mg/L), and France (8 mg/L)] [10,11].

To date, few scientists have used HPLC with pre-column derivatization and UV detection to analyze BAs contents in 53 Chinese soy sauces. Some results showed that only the levels of 5 kinds of BAs were quantified [12]. The aim of this study is to extensively determine the types and concentrations of the major BAs in some soy sauces from different regions and manufacturers in China using an improved HPLC method with higher sensitivity.

## 2. Results and Discussion

### 2.1. Method Evaluation for Biogenic Amines Analysis

**Figure 1:**
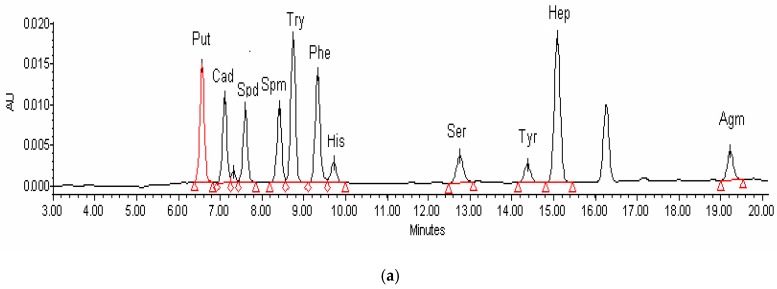

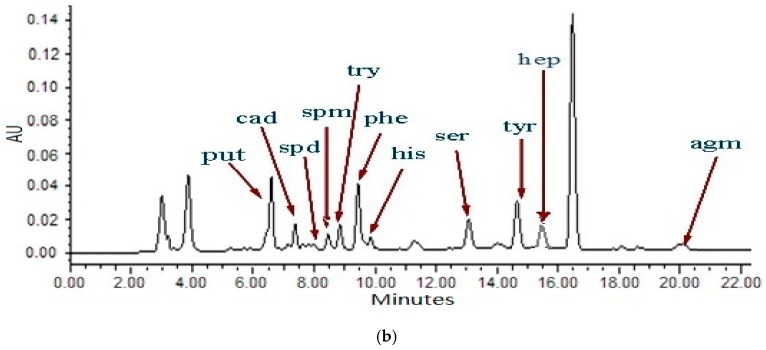
Chromatogram of derivatives of BA standards (5 μg /mL) (**a**) and BAs in soy sauce (**b**).

The ten biogenic amines and the internal standard were all well resolved with the gradient elution pattern described in Table 1. Figure 1a,b showed typical chromatograms of biogenic amines in the standard solutions and the Chinese soy sauce samples, respectively. Compared with the retention time of the standard solutions, biogenic amines could be identified clearly.

Figure 1a showed the chromatogram of ten BAs standards. It can be observed that both different BAs (5μg/mL) and the internal standard derivatives were effectively separated. Besides, the samples were quickly eluted, and all tested derivatives were also successfully eluted within 20 min. Figure 1b exhibited the chromatogram of derivatives of soy sauce sample (sample 1) extract. The peak shape of different BAs was symmetrical without impurity interference. This suggested that the mobile phase elution gradient met the requirement of simultaneous determination of the ten BAs.

### 2.2. Method Validation

The mixed standard solutions (0.1, 0.5, 1, 5, 10, 20, 50, 80, 100 μg/mL, respectively) were injected into the HPLC detection system in sequence. The regression equation and correlation coefficient of the standard curves were calculated according to the ratio of the peak area to the internal standard peak area (Table 2). The concentration of corresponding substances in the samples were determined by the peak area ratio. The acquired linear correlation coefficient of each bioamine standard was greater than 0.99 with detection limits of 0.01–0.03 μg/mL and quantitation limit of 0.03–0.10 μg/mL. This demonstrated that the method built possessed high sensitivity and can meet the requirement of the soy sauce samples during determination.

### 2.3. BAs Contents in Chinese Commercial Soy Sauces

The determination results of different BA contents in several Chinese soy sauce brands are shown in Table 3. Put, Cad, Spd, Spm, Try, Phe, His, Tyr, Ser, and Agm were found in the most of Chinese soy sauce samples. However, their contents varied significantly in the various soy sauces (the different brands, districts and types). The content scopes of Put, Cad, Spd, Spm, Try, Phe, His, Tyr, Ser, Agm, and total BAs in the soy sauce samples were ND (not detected)–44.59mg/L, ND–82.36 mg/L, ND–63.47mg/L, ND–17.17 mg/L, ND–124.34 mg/L, ND–263.79 mg/L, ND–477.79 mg/L, 3.50–15.75 mg/L, 4.13–257.38 mg/L, 0.55–34.57 mg/L, and 45.63–611.25 mg/L, respectively. This suggested that there were marked differences between the contents of BAs in each sample. Besides, the results showed that the average contents of Tyr, Phe, and Put were the highest in the soy sauce. This conform with a previous report [13]. Also, the different processing environments of the soy sauces played an important role in the synthesis of BAs. In addition, there were significant differences in the content of BAs between the dark soy and the light soy sauces, which indicated that the differences in processing techniques or the fermentation strains used was one of the key factors that caused the varieties of BAs contents in Chinese soy sauces. Similar results were reported elsewhere [2,14,15,16]. 

**Figure 2:**
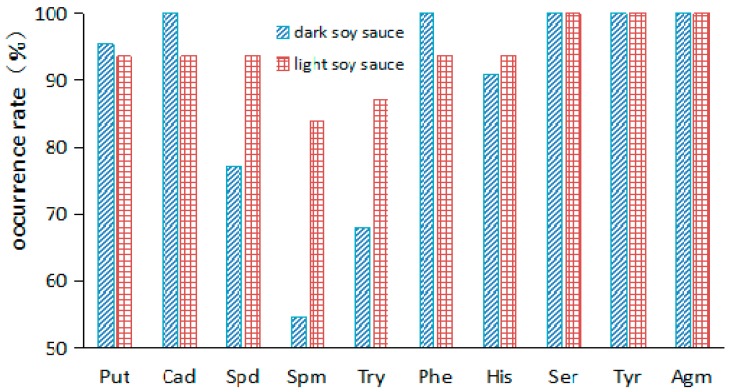
Occurrence of BAs in two kinds of soy sauces.

It could be seen that at least six types of BAs were detected in each Chinese soy sauce sample (Table 3). Ser, Tyr, and Agm were the most commonly detected in both dark and light soy sauce samples (Figure 2). In addition, the occurrence rates of Put, Cad, and Phe in the dark soy sauce samples were higher than that in the light ones. However, Spd, Spm, Ser, and His showed the opposite trend. That is the occurrence rates of these BAs in dark soy sauce samples were lower than those in the light samples. Similar results about the occurrence rates of Put, Tyr and His, as well as Cad and Phe were reported [13,16,17].

In all the Chinese soy sauce samples tested, His, Tyr, and Phe were the highest contents recorded and this corresponded to 477.79, 257.38, and 263.79 mg/L, respectively. This was similar with previous results [16]. A researcher analyzed five BAs in soy sauce produced from different locations (Guangdong, Shanghai and Jiangsu), and found that four types of the samples produced from Guangzhou had the highest histamine content of 592 ± 0.43 mg/L [12].

**Figure 3:**
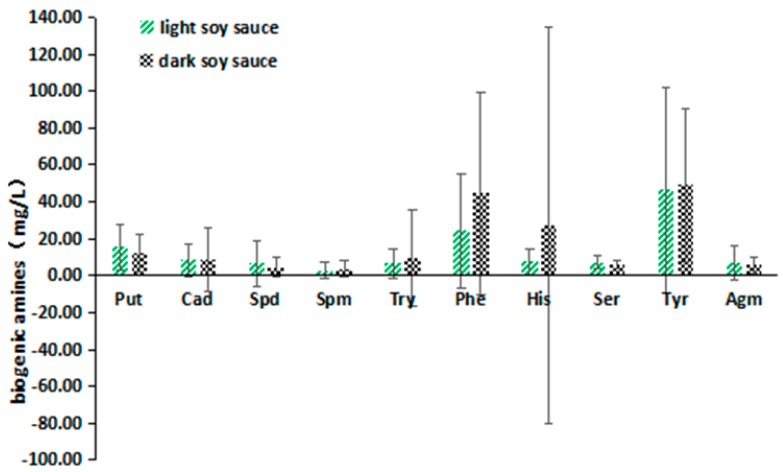
The contents of BAs in different types of Chinese soy sauces.

As shown in Figure 3, the average contents of Cad, Spm, Try, Phe, His and Tyr in dark soy sauce samples were more than those in light soy sauce samples. Especially for His, the most harmful biogenic amine, its average concentration in dark soy sauce samples was 3.7 times higher than that in the light soy sauce samples. Likewise, we can find that the average concentration of Phe in the dark soy sauces was 1.84 times higher than that in light soy sauces. Besides, the other eight kinds of BAs differed considerably in different types of Chinese soy sauces. Furthermore, the large error bars illustrated the differences in every BA content in the same type of soy sauces.

**Figure 4:**
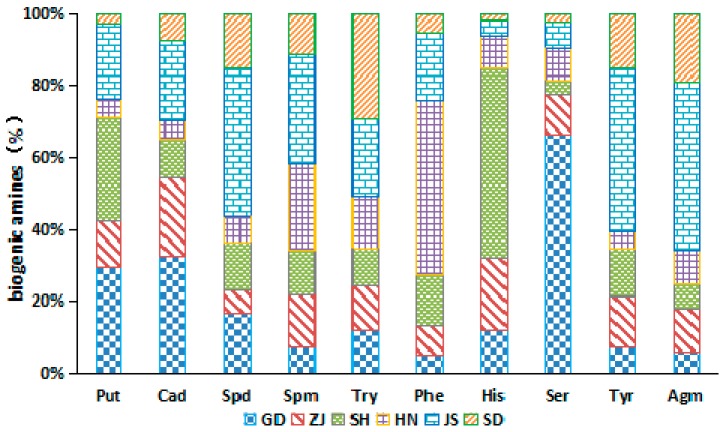
The average percentages of biogenic amines in Chinese soy sauce samples from different production regions.

The various kinds of the ten BA contents in each Chinese soy sauces were related to their production places (Figure 4). There was no significant difference in the contents of the ten biogenic amines obtained in Zhejiang soy sauce. This suggested that the concentration of each BA was similar in this soy sauce. However, the concentrations of Put, Cad, and Ser were higher than the other BAs in the soy sauce from Guangdong. On the other hand, higher contents of Put and His were found in Shanghai soy sauce. Whereas Spd, Spm, Tyr, and Agm were prevalent in all the BAs in Jiangsu soy sauces. Also, Try and Ser were the most BA components recorded in Hunan and Shandong samples, respectively. The main reason the BA categories and contents were different in the soy sauce produced from the different regions might be due to the differences in processing technology and environment [18].

Although the highest single dose of His and Tyr contents in 53 Chinese soy sauce samples was lower than the His and Tyr toxicological limits, the His concentration in S30 sample was 477.79 mg/kg, which was more than the maximum levels recommended in aquatic products by the US FDA and European food safety authority (50 mg/kg and 400 mg/kg, respectively) [19]. Furthermore, previous reports revealed that Try and His were synergistically toxic to intestinal cells in culture. Results showed that 190 mg/kg of His, a concentration below the restrictive legal limit (200 mg/kg), could increase the toxicity when mixed with 500 mg/kg of tyramine (a concentration easily reached in some foods) [20]. Therefore, considering this synergistic toxicity between the different BAs, the content of each biogenic amine in soy sauces should be subjected to stricter legal regulation.

## 3. Materials and Methods

### 3.1. Samples, Reagents and Instrument

A total of 53 traditional Chinese soy sauce samples were acquired from various supermarkets in Hangzhou, China. All the collected samples were stored at 4 °C until analysis.

All chemicals used were of chromatographic grade. Try, Phe, His, Tyr, Put, Cad, Agm, Spd, Spm, Hep and benzoyl chloride (Dns-Cl) were purchased from Sigma–Aldrich Co. Ltd. (St. Louis, MO, USA), and serotonin (Ser) was acquired from Alfa Co. Ltd. (Binfield, Bracknell, UK). Acetone for HPLC was obtained from Tedia Co. Inc. (Fairfield, OH, USA). 

Ten target BAs were separated and quantified using a Waters 1525 HPLC system (Waters Co., Milford, MA, USA) with a Waters 2487 dual wavelength absorbance UV detector ((Waters Co., Milford, MA, USA). Inertsil ODS-3 column (250*4.6 mm, 5 mm, GL Science Inc., Tokyo, Japan) with a Hypersil ODS-3 guard column was also used to detect the BAs.

### 3.2. Determination of BAs

#### 3.2.1. Preparation of BA Standard Solutions

Ten BA standard solutions were prepared from a 1 mg/mL stock solution based on their effective concentration using 0.1 mol/L hydrochloric acid (HCl) solution. The 100 μg/mL working solution of mixed standards was acquired by mixing the same content of the ten BAs. The 1 mg/mL stock solution of internal standard heptamine (Hep) was dissolved in 0.1 mol/L HCl solution to prepare 100 μg/mL internal working solution. All solutions were kept in the dark at 4 °C.

#### 3.2.2. Standard Curve

Standard curve drawing was drawn according to a previously described method [21,22,23]. The standard solution was diluted to different concentrations (80, 50, 20, 10, 5, 1, 0.5, and 0.1 μg/mL) with 0.1 mol/L HCl. The experiment was carried out as followed: 100 μL of internal standard working solution (100 μg/mL), 1 mL of NaOH (2 mol/L) and 10 μL of Dns-Cl were added to 2 mL of diluted solutions of different concentrations, after which the above solution was well vortexed and then put in the water bath for 50 min of reaction at 30 °C. An aliquot of 2 mL of saturated NaCl solution was used to terminate the reaction after 30 s of vortexing. This was followed by the addition of 3 mL of anhydrous ether twice separately for extraction. The mixed solution was centrifuged at 1200 rpm for 4 min and the ether layer was removed into a glass tube and dried with nitrogen. After that, the dried product was dissolved in 1 mL of acetonitrile and filtered with 0.45 μm polyvinylidine difluoride filter (PVDF) for subsequent determination. Three parallels were built for each sample. All samples were stored at 4°C in the refrigerator and detected within 24 h.

#### 3.2.3. Sample Pretreatment

About 20mL of perchloric acid solution and 100 μL of Hep solution were added to 2mL of soy sauce samples, followed by oscillation for 5 min and allowed to stand for another 5 min. Afterward, 10 µL of NaOH (2 mol/L) and 10 µL of Dns-Cl were added to 1mL of the mixture, followed by oscillation and reaction in a water bath at 30 °C for 40 min. Besides, 2 mL of saturated NaCl was added and then oscillated for 10 s. After that, 3 mL of anhydrous ether was added for extraction before rolling-over shaking for 5 s and centrifugation at 1000 rpm for 5 min. The extraction steps were performed three times, and the accumulated upper ether layers acquired were removed into a centrifuge tube for drying with nitrogen. Finally, the dried product was added to 1 mL of acetonitrile. This mixture was vortexed thoroughly and filtered with 0.45 μm PVDF for subsequent analysis. Three parallels were established for each sample.

#### 3.2.4. HPLC Detection

For BAs measurement, a Waters HPLC apparatus equipped with a UV detector was used. The column was ODS-3 column (5 μm, 250 × 4.6mm) and the column temperature was 30 °C. The UV detection was 254 nm. Chromatographic separation was carried out using continuous gradient elution with 0.005 mol/L ammonium acetate solution (eluent A) and HPLC grade acetonitrile (eluent B). The gradient elution methods of mobile phases are listed in Table 1. The flow rate of performed elution was 1.0 mL/min. The total separation time was less than 20 min, and the injection volume was 20 μL.

In addition, the derivatives of mixed standards with a concentration of 0.1 μg/mL were appropriately diluted with acetonitrile. Signal-to-noise ratio (S/N) of greater than 3 was regarded as the criterion for detection limits, and S/N of greater than 10 as the criterion for quantitation limits.

### 3.3. Statistical Analysis

SPSS 20.0 software was used to perform all statistical analyses. All data were expressed as means ± SD (n = 3).

## 4. Conclusions

In the current study, the contents of ten important BAs in 53 Chinese commercial soy sauces were systematically compared. Put, Cad, Spd, Spm, Try, Phe, His, Tyr, Ser and Agm were detected in most of the samples. Results showed that there were significant differences in the total concentration of BAs in all the commercial soy sauces. Besides, the proportion of each kind of biogenic amine also varied dramatically in the soy sauce samples with different brands and processing technology from different production places. The average contents of Cad, Spm, Try, Phe, His and Tyr in dark soy sauce samples were much higher compared to those found in the light samples. Similarly, the average concentration gaps of His and Phe between the dark and the light soy sauces were 3.7 and 1.8 times, respectively. Furthermore, we discovered that the production regions played a significant role in the BA concentration in the commercial soy sauces mainly due to different processing technology and processing environment. Based on the results of this work, we can study the production mechanism and control process of BAs in the future.

## Figures and Tables

**Table 1:** Gradient elution program for biogenic amines analysis.

Time (min)	0	2	5	10	16	20	21	25	27	36
Acetonitrile (%)	60	60	38	38	20	20	0	0	60	60
Water (%)	40	40	62	62	80	80	100	100	40	40

**Table 2:** Linear ranges, calibration curves, correlation coefficient (R), method limits of detection and quantitation (MLODs and MLOQs) of ten BAs.

BA	Calibration Curves	R^2^	Linearity Range (μg/mL)	MLODs (μg/mL)	MLOQs (μg/mL)
Put	y = 9.6368x − 0.5572	0.9969	0.1–80	0.01	0.03
Cad	y = 15.548x − 0.6264	0.9980	0.1–80	0.01	0.04
Spd	y = 13.048x − 0.5076	0.9951	0.1–80	0.01	0.04
Spm	y = 14.387x − 0.6906	0.9935	0.1–80	0.01	0.03
Tyr	y = 15.981x − 0.6751	0.9971	0.1–80	0.01	0.04
Phe	y = 19.275x − 0.9176	0.9905	0.1–80	0.01	0.03
His	y = 12.766x − 0.0525	0.9981	0.1–80	0.03	0.10
Ser	y = 9.0984x + 1.6702	0.9909	0.1–80	0.03	0.10
Try	y = 22.662x − 0.1399	0.9901	0.1–80	0.03	0.09
Agm	y = 10.027x + 0.2399	0.9934	0.1–80	0.03	0.10

Note: In the Calibration curves, x represents the ratio of the standard peak area to the internal standard peak area; y represents the concentration of standard solutions (μg/mL).

**Table 3:** Biogenic amine content in Chinese soy sauce from the market.

Samples		Biogenic Amines^m^										
		Put	Cad	Spd	Spm	Try	Phe	His	Ser	Tyr	Agm	Total
s1	GD^a^_x_	16.63 ± 0.60	3.18 ± 0.28	2.50 ± 0.25	5.34 ± 0.47	11.40 ± 1.24	64.70 ± 5.30	4.50 ± 0.64	3.72 ± 0.06	14.09 ± 1.56	1.80 ± 0.58	127.84 ± 8.63
s2	GD_y_	29.76 ± 1.11	4.58 ± 0.60	2.27 ± 0.72	7.65 ± 1.37	12.59 ± 0.95	68.10 ± 3.67	4.56 ± 0.34	3.55 ± 0.05	9.03 ± 0.28	1.41 ± 0.63	143.49 ± 7.69
s3	GD_y_	3.23 ± 0.13	1.72 ± 0.08	3.17 ± 0.11	1.29 ± 0.08	2.40 ± 0.19	10.66 ± 0.63	2.68 ± 0.25	4.74 ± 0.15	31.35 ± 2.51	4.11 ± 0.23	65.36 ± 3.95
s4	HN^b^_x_	2.02 ± 0.16	ND^n^	2.13 ± 0.10	8.92 ± 0.59	8.98 ± 0.86	26.51 ± 1.41	1.64 ± 0.78	8.93 ± 0.99	31.11 ± 1.86	6.41 ± 1.34	96.64 ± 1.22
s5	HN_x_	8.68 ± 1.13	ND	ND	17.17 ± 1.09	124.34 ± 8.83	24.81 ± 2.15	1.93 ± 0.12	9.65 ± 0.37	34.22 ± 0.42	6.89 ± 1.04	227.69 ± 9.62
s6	HN_y_	0.97 ± 0.32	5.05 ± 0.69	6.17 ± 0.97	2.58 ± 0.22	7.30 ± 0.74	7.22 ± 1.22	12.42 ± 1.22	8.76 ± 0.77	14.09 ± 1.79	7.31 ± 0.51	71.87 ± 4.40
s7	HN_y_	0.67 ± 0.02	2.55 ± 0.18	3.65 ± 0.45	ND	9.11 ± 0.55	5.59 ± 0.41	1.40 ± 0.29	3.97 ± 0.43	12.44 ± 0.14	6.25 ± 0.55	45.63 ± 0.53
s8	GD_y_	10.83 ± 0.33	1.93 ± 0.04	1.78 ± 0.34	0.98 ± 0.19	4.81 ± 0.52	38.43 ± 2.62	15.09 ± 0.04	4.78 ± 0.21	10.86 ± 1.02	2.19 ± 0.08	91.67 ± 2.85
s9	GD_x_	25.45 ± 1.20	18.68 ± 1.45	8.83 ± 0.08	4.15 ± 0.12	2.88 ± 0.02	37.08 ± 2.84	198.35 ± 4.85	9.26 ± 0.06	85.75 ± 1.99	19.17 ± 1.05	409.60 ± 9.00
s10	GD_y_	33.92 ± 0.27	2.58 ± 0.29	0.61 ± 0.12	ND	6.98 ± 0.80	35.56 ± 2.99	11.69 ± 0.74	4.14 ± 0.25	5.01 ± 0.10	0.79 ± 0.11	101.27 ± 5.66
s11	GD_y_	34.50 ± 0.31	3.94 ± 0.30	ND	ND	6.71 ± 0.36	31.50 ± 2.41	13.37 ± 0.92	3.87 ± 0.02	4.64 ± 0.05	0.55 ± 0.02	99.07 ± 3.63
s12	GD_y_	38.77 ± 1.13	7.43 ± 0.22	0.65 ± 0.02	ND	8.36 ± 1.40	62.93 ± 4.03	12.59 ± 1.14	3.55 ± 0.04	4.13 ± 0.07	0.78 ± 0.03	139.16 ± 7.98
s13	GD_y_	25.57 ± 0.54	1.59 ± 0.12	1.47 ± 0.02	ND	3.25 ± 0.21	15.72 ± 0.86	6.84 ± 0.09	5.50 ± 0.06	13.66 ± 0.48	1.72 ± 0.09	75.31 ± 1.82
s14	JS^c^_y_	18.43 ± 0.97	13.82 ± 1.27	27.91 ± 0.96	14.68 ± 0.96	29.12 ± 1.22	12.77 ± 0.98	4.59 ± 0.18	11.81 ± 1.05	158.63 ± 6.37	34.57 ± 1.59	326.33 ± 13.09
s15	JS_y_	7.73 ± 0.87	2.23 ± 0.32	5.32 ± 0.14	3.39 ± 0.34	ND	2.31 ± 0.05	2.17 ± 0.13	11.47 ± 0.45	257.38 ± 13.31	32.16 ± 1.37	324.16 ± 14.76
s16	ZJ^d^_y_	ND	19.04 ± 0.71	1.94 ± 0.01	ND	ND	ND	12.80 ± 0.38	9.18 ± 0.05	54.02 ± 0.32	7.03 ± 0.23	104.49 ± 0.34
s17	ZJ_y_	10.26 ± 0.84	9.94 ± 0.63	6.78 ± 0.22	10.86 ± 1.42	21.45 ± 0.69	7.77 ± 0.23	3.12 ± 0.18	7.94 ± 0.52	87.05 ± 5.16	24.42 ± 1.06	189.59 ± 2.97
s18	ZJ_y_	8.65 ± 0.42	8.52 ± 0.64	3.51 ± 0.77	9.50 ± 0.94	23.37 ± 2.14	5.98 ± 0.46	2.64 ± 0.51	10.11 ± 0.95	104.07 ± 7.14	24.39 ± 2.71	200.74 ± 12.86
s19	GD_y_	13.69 ± 0.33	2.73 ± 0.11	0.54 ± 0.07	1.12 ± 0.21	3.19 ± 0.11	11.43 ± 0.56	8.08 ± 0.97	8.61 ± 0.66	44.10 ± 4.85	6.40 ± 0.22	99.90 ± 8.08
s20	GD_x_	23.04 ± 1.17	16.20 ± 0.68	2.86 ± 0.17	ND	3.38 ± 0.34	66.85 ± 6.26	4.39 ± 0.31	4.36 ± 0.67	29.07 ± 2.42	3.31 ± 0.23	153.78 ± 8.51
s21	GD_y_	14.88 ± 0.01	3.41 ± 0.10	1.32 ± 0.03	ND	1.92 ± 0.01	10.71 ± 0.32	6.83 ± 0.03	4.16 ± 0.01	12.30 ± 0.08	1.84 ± 0.20	57.76 ± 0.07
s22	GD_y_	6.27 ± 0.34	3.88 ± 0.34	5.59 ± 0.29	ND	0.73 ± 0.06	5.70 ± 0.11	1.88 ± 0.06	8.88 ± 0.16	28.90 ± 0.35	1.76 ± 0.10	63.84 ± 0.79
s23	GD_y_	4.12 ± 0.22	4.70 ± 0.21	4.60 ± 0.28	1.22 ± 0.10	ND	2.06 ± 0.06	2.87 ± 0.09	8.39 ± 0.06	28.52 ± 0.65	2.88 ± 0.47	59.37 ± 0.21
s24	GD_y_	4.64 ± 0.00	4.62 ± 0.44	6.34 ± 0.33	1.60 ± 0.10	ND	6.25 ± 0.06	1.49 ± 0.02	9.10 ± 0.02	32.09 ± 0.26	2.62 ± 0.12	69.24 ± 0.13
s25	GD_x_	22.41 ± 1.00	82.36 ± 3.75	21.69 ± 0.33	5.61 ± 0.13	4.57 ± 0.05	20.38 ± 1.05	5.25 ± 0.73	7.13 ± 0.08	40.41 ± 1.16	9.65 ± 0.20	219.46 ± 1.77
s26	GD_x_	16.10 ± 0.84	5.17 ± 0.47	ND	ND	ND	ND	13.53 ± 1.12	6.59 ± 0.64	79.57 ± 5.26	5.73 ± 0.30	126.69 ± 6.95
s27	SD^e^_y_	1.62 ± 0.04	2.73 ± 0.04	6.08 ± 0.07	3.40 ± 0.26	3.99 ± 0.28	3.02 ± 0.70	1.08 ± 0.13	15.75 ± 0.57	68.78 ± 4.02	13.57 ± 0.79	120.02 ± 4.57
s28	GD_y_	14.69 ± 1.08	9.39 ± 0.03	0.65 ± 0.11	0.93 ± 0.30	1.95 ± 0.46	9.99 ± 0.04	12.69 ± 1.27	7.85 ± 0.31	13.99 ± 0.35	2.82 ± 0.40	74.95 ± 2.93
s29	GD_x_	4.83 ± 0.23	3.56 ± 0.15	3.80 ± 0.39	2.64 ± 0.23	5.16 ± 0.26	2.40 ± 0.52	1.04 ± 0.03	7.58 ± 0.31	38.61 ± 1.72	5.51 ± 0.92	75.15 ± 2.11
s30	GD_x_	11.72 ± 0.92	5.67 ± 0.60	3.96 ± 0.65	3.77 ± 0.05	1.49 ± 0.07	4.09 ± 1.16	477.79 ± 23.52	9.77 ± 0.28	85.54 ± 5.37	7.46 ± 0.95	611.25 ± 22.13
s31	GD_y_	37.80 ± 1.98	29.55 ± 1.32	63.47 ± 4.73	2.58 ± 0.24	8.08 ± 0.86	20.15 ± 1.27	36.36 ± 0.40	13.85 ± 0.35	70.66 ± 1.29	5.56 ± 0.78	288.08 ± 4.32
s32	GD_x_	12.23 ± 0.99	24.17 ± 1.11	13.55 ± 0.40	3.89 ± 0.43	ND	3.29 ± 0.75	5.06 ± 0.21	8.61 ± 0.12	61.82 ± 3.96	6.26 ± 0.48	138.87 ± 2.97
s33	GD_y_	18.77 ± 0.58	8.53 ± 0.04	ND	ND	1.23 ± 0.08	3.94 ± 0.19	12.20 ± 0.61	3.50 ± 0.03	9.53 ± 0.84	0.70 ± 0.09	58.66 ± 2.15
s34	GD_y_	39.09 ± 1.73	28.09 ± 0.31	21.59 ± 0.80	1.52 ± 0.39	4.81 ± 0.44	5.86 ± 0.33	6.25 ± 0.14	8.15 ± 0.20	43.41 ± 1.31	4.84 ± 0.18	163.61 ± 5.46
s35	GD_x_	ND	5.89 ± 0.32	5.08 ± 0.16	1.29 ± 0.00	1.90 ± 0.11	2.03 ± 0.24	1.54 ± 0.15	5.42 ± 0.04	31.54 ± 0.59	4.78 ± 0.46	59.93 ± 0.42
s36	GD_x_	15.09 ± 0.46	8.96 ± 0.22	0.86 ± 0.07	1.48 ± 0.06	1.85 ± 0.07	19.50 ± 0.06	15.86 ± 0.77	3.92 ± 0.08	10.30 ± 1.61	2.09 ± 0.15	79.90 ± 1.93
s37	GD_y_	29.84 ± 1.92	31.18 ± 1.92	8.41 ± 0.34	2.78 ± 0.75	ND	11.83 ± 0.54	1.07 ± 0.27	8.29 ± 0.25	75.93 ± 5.92	5.65 ± 0.51	174.96 ± 11.74
s38	ZJ_y_	14.41 ± 1.89	29.31 ± 0.10	5.98 ± 1.19	14.04 ± 0.53	1.50 ± 0.33	12.12 ± 1.13	5.71 ± 0.50	11.89 ± 1.06	144.55 ± 0.89	12.30 ± 0.68	251.81 ± 2.73
s39	ZJ_x_	5.97 ± 0.82	6.24 ± 0.60	1.72 ± 0.15	1.69 ± 0.31	ND	7.86 ± 0.68	6.98 ± 0.64	5.04 ± 0.01	26.24 ± 1.68	3.33 ± 0.26	65.29 ± 5.05
s40	ZJ_x_	13.03 ± 0.42	4.98 ± 0.25	ND	1.52 ± 0.22	4.05 ± 0.30	81.02 ± 4.48	7.48 ± 0.82	3.80 ± 0.07	16.40 ± 0.29	3.63 ± 0.21	136.26 ± 6.39
s41	ZJ_x_	7.08 ± 0.26	3.58 ± 0.01	ND	6.93 ± 0.85	9.97 ± 0.02	20.85 ± 0.51	0.82 ± 0.14	4.97 ± 0.20	59.01 ± 0.13	9.05 ± 0.07	122.27 ± 2.20
s42	ZJ_y_	3.74 ± 0.58	2.52 ± 0.70	ND	4.59 ± 0.58	ND	40.53 ± 2.37	ND	7.38 ± 0.89	89.20 ± 8.68	4.63 ± 0.85	152.93 ± 14.75
s43	ZJ_y_	5.81 ± 0.08	3.25 ± 0.03	0.63 ± 0.14	1.19 ± 0.04	ND	23.64 ± 0.57	1.40 ± 0.14	5.20 ± 0.08	42.47 ± 1.89	0.79 ± 0.13	84.38 ± 1.32
s44	ZJ_x_	12.63 ± 0.53	8.95 ± 1.22	9.27 ± 1.75	13.73 ± 0.29	11.11 ± 0.64	41.05 ± 0.28	4.78 ± 0.17	9.83 ± 0.44	167.02 ± 1.88	15.92 ± 0.26	294.29 ± 3.44
s45	ZJ_x_	17.84 ± 0.07	4.41 ± 0.03	4.80 ± 0.22	2.41 ± 0.37	10.76 ± 0.60	67.80 ± 2.59	4.60 ± 0.23	3.86 ± 0.33	21.91 ± 1.33	3.74 ± 0.74	142.12 ± 1.46
s46	ZJ_y_	44.59 ± 1.53	2.13 ± 0.27	10.41 ± 1.20	ND	20.18 ± 1.43	263.79 ± 16.14	ND	4.02 ± 0.14	17.96 ± 0.48	2.33 ± 0.15	365.41 ± 19.54
s47	SH^f^_x_	16.87 ± 0.56	2.85 ± 0.54	3.13 ± 0.59	ND	5.67 ± 0.13	88.10 ± 1.27	7.97 ± 0.27	3.89 ± 0.17	19.02 ± 1.16	2.64 ± 0.09	150.55 ± 2.25
s48	SH_x_	12.95 ± 0.03	1.36 ± 0.59	1.27 ± 0.10	ND	5.67 ± 0.84	96.24 ± 4.63	14.43 ± 0.70	3.62 ± 0.02	6.63 ± 0.49	1.35 ± 0.26	143.53 ± 7.55
s49	SH_x_	2.52 ± 0.05	2.62 ± 0.06	4.68 ± 0.49	2.99 ± 0.27	1.63 ± 0.36	32.35 ± 2.02	ND	5.86 ± 0.32	92.08 ± 7.45	8.24 ± 0.63	152.96 ± 6.42
s50	SH_x_	3.45 ± 0.08	2.99 ± 0.07	2.22 ± 0.23	4.34 ± 0.98	0.61 ± 0.01	42.79 ± 1.22	0.61 ± 0.41	8.11 ± 0.55	137.41 ± 4.72	4.81 ± 0.84	207.34 ± 7.15
s51	SH_x_	ND	3.84 ± 0.02	0.96 ± 0.15	0.69 ± 0.04	ND	28.47 ± 0.41	2.73 ± 0.07	5.23 ± 0.15	27.73 ± 2.41	1.18 ± 0.12	70.83 ± 2.41
s52	SH_y_	21.31 ± 2.05	2.20 ± 0.39	6.60 ± 0.37	ND	24.02 ± 3.96	119.46 ± 4.20	ND	4.09 ± 0.04	20.12 ± 1.18	2.04 ± 0.24	199.85 ± 12.36
s53	GD_y_	1.55 ± 0.28	6.29 ± 0.12	2.15 ± 0.14	8.09 ± 0.98	9.62 ± 0.11	11.93 ± 0.18	4.00 ± 0.01	5.06 ± 0.07	33.02 ± 0.33	7.01 ± 0.55	88.72 ± 1.02

^a–f^: GD, HN, JS, ZJ, SD and SH mean Guangdong, Hunan, Jiangsu, Zhejiang, Shandong and Shanghai, respectively; _x-y_: _x_ means dark soy sauce, _y_ means light soy sauce; ^m^: n = 3, mg/L, wet weight basis; ^n^: ND, not detected (Put, Cad, Spd, Spm, Try, Phe, His, Ser, Tyr and Agm < 0.5 mg/kg, wet weight basis).
